# A Korean child diagnosed with malonic aciduria harboring a novel start codon mutation following presentation with dilated cardiomyopathy

**DOI:** 10.1002/mgg3.1379

**Published:** 2020-06-30

**Authors:** Seung Hoon Lee, Jung Min Ko, Mi‐Kyoung Song, Junghan Song, Kyung Sun Park

**Affiliations:** ^1^ Department of Pediatrics Seoul National University College of Medicine Seoul Korea; ^2^ Department of Laboratory Medicine Seoul National University College of Medicine Seoul Korea; ^3^ Department of Laboratory Medicine Kyung Hee University School of Medicine Seoul Korea

**Keywords:** dilated cardiomyopathy, malonic aciduria, *MLYCD*, newborn screening

## Abstract

**Background:**

Malonic aciduria (MA, OMIM#248360) is an extremely rare inherited metabolic disorder caused by the deficiency of malonyl‐CoA decarboxylase. The phenotype exhibited by patients with MA is variable, but may include symptoms, such as developmental delay in early childhood, seizures, vomiting, metabolic acidosis, hypoglycemia, ketosis, and cardiomyopathy. We describe the first case of a Korean child with MA who presented with dilated cardiomyopathy (DCMP) at the age of 3 months.

**Methods and Results:**

A 3‐month‐old Korean boy visited our hospital for diagnosis and management of cardiomegaly. Newborn screening for inherited metabolic diseases showed a normal result; therefore, DCMP management was initiated. Biochemical and the *MLYCD* gene analyses subsequently confirmed diagnosis of MA. Elevated plasma C3DC level and excessive excretion of urinary malonate were observed, and two pathogenic variants, including a novel start codon mutation (c.1A>G), were identified in *MLYCD*. A low long‐chain fat diet with middle‐chain triglyceride formula and L‐carnitine supplementation was initiated. The patient is now 5 years old and exhibits considerably improved cardiac function.

**Conclusions:**

MA can be diagnosed using newborn screening; however, negative results do not exclude the possibility of disease. Metabolic screening for differential diagnosis of infantile DCMP is recommended to rule out rare, but manageable, metabolic cardiomyopathies.

## Graphical abstract

This is the first report of MA diagnosed in a patient following presentation with infantile‐onset dilated cardiomyopathy following negative malonylcarnitine measurement results in a newborn screening program. The disease was successfully managed with a combination of medical therapy and dietary intervention.

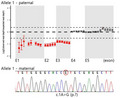

## 1 INTRODUCTION

Malonic aciduria (MA, OMIM#248360) is an extremely rare genetic disorder caused by the deficiency of malonyl‐CoA decarboxylase (MCD). Since being first described in 1984 (Brown, Scholem, Bankier, & Danks, 1984), less than 40 cases of MCD deficiency have been reported in the literature (Liu et al., 2016). Following a report in the early 2000s, which showed that malonylcarnitine (C3DC) in the blood could be measured using tandem mass spectrometry (TMS) (Santer et al., 2003), there have been several case reports of MA being diagnosed worldwide using the technique during the asymptomatic neonatal period.

MCD, which catalyzes the conversion of malonyl‐CoA to acetyl‐CoA and carbon dioxide, is encoded by the *MLYCD* gene (OMIM*606761) located on chromosome 16q23. The enzyme is expressed in heart, skeletal muscle, liver, kidneys, pancreas, and brain. MCD deficiency results in accumulation of malonyl‐CoA in the cytoplasm, which in turn inhibits carnitine palmitoyltransferase 1 (McGarry & Brown, 1997), the rate‐limiting enzyme in fatty acid beta‐oxidation in mitochondria and peroxisomes. The characteristic phenotype is highly variable, and includes developmental delay, seizures, diarrhea, vomiting, metabolic acidosis, hypoglycemia, ketosis, and cardiomyopathy (Celato et al., 2013). Cardiomyopathy is a leading cause of mortality and morbidity in MA, and approximately first/third of the patients with MA show cardiomyopathy during the clinical course of the disease (Celato et al., 2013). A high middle‐chain triglyceride (MCT)/low long‐chain triglyceride diet combined with a carnitine supplement could be an effective method for preserving cardiac function in patients with MA (Ficicioglu, Chrisant, Payan, & Chace, 2005; Footitt et al., 2010).

Here, we describe the first case of a Korean child with MA who presented with dilated cardiomyopathy (DCMP) at the age of 3 months. Diagnosis was confirmed using biochemical and molecular analyses.

## 2 MATERIALS AND METHODS

### 2.1 Ethical compliance

The Institutional Review Board of Seoul National University Hospital approved this study (H‐2001‐153‐1099). The study was performed in accordance with the Declaration of Helsinki and written informed consent for molecular study and publication was obtained from the patient's parents.

### 2.2 Clinical report and results of biochemical analyses

A 3‐month‐old Korean boy was transferred to our hospital for diagnosis and management of progressive cardiomegaly. He was the first child born to healthy and nonconsanguineous parents at 37 + 2 weeks of gestation with a birth weight of 2.68 kg (10–50th percentile). Immediately after birth, he was admitted to the neonatal intensive care unit (NICU) for 8 days owing to tachypnea and cardiac murmur. Echocardiography revealed normal cardiac function at that time. A newborn screening test using TMS showed a normal result on the third day after birth. During the follow‐up period at an out‐patient clinic, his oral intake was reported to have slowly decreased and his cardiomegaly progressed with an ejection fraction of 20% (reference range: 56%–78%). Management for congestive heart failure with digoxin was initiated 2 weeks before admission to our hospital.

When he arrived at our hospital, his vital signs were normal, with the exception of mild tachypnea. His height and weight were 62.8 cm (50–75th percentile) and 6.2 kg (10–25th percentile), respectively, and his head circumference was 42 cm (75–90th percentile). He was not dysmorphic or hypotonic and exhibited no other noticeable abnormalities in a physical examination. Similarly, no cardiac structural anomalies were detected in the follow‐up echocardiographic examination. However, his ventricular septum was hypokinetic and showed paradoxical movement. The patient exhibited fractional shortening of 13% (reference range: 28%–46%) and an ejection fraction of 28%.

With regard to laboratory findings, metabolic acidosis was absent on the venous blood gas analysis. The plasma level of ammonia was normal and no ketone bodies were detected in urine sample. Acylcarnitine analysis using TMS showed an elevated C3DC level of 1.61 µmol/L (reference value <0.36 µmol/L). Organic acid analysis of the urine showed excessive excretion of malonate (815.04 mmol/mol Cr; reference value, not detected) and methylmalonate (146.38 mmol/mol Cr; reference value <3.6 mmol/mol Cr), suggesting MA (Table 1).

**Table 1:** Results of the biochemical analyses performed

	Measured value	Reference value
Plasma		
Malonylcarnitine (C3DC)	1.61 µmol/L	<0.36 µmol/L
Free carnitine	8.0 µmol/L	28.0–52.0 µmol/L
Acylcarnitine, total	3.9 µmol/L	7.0–22.1 µmol/L
Urine		
Malonate	815.04 mmol/mol Cr	Not detected
Methylmalonate	146.38 mmol/mol Cr	<3.6 mmol/mol Cr

L‐carnitine replacement (100 mg kg^‐1^ day^‐1^), high‐carbohydrate/low‐fat diet, and MCT supplementation was started. Following 3 weeks of treatment, an echocardiogram showed improvement in systolic function with fractional shortening of 21% and an ejection fraction of 42% although plasma C3DC level was still elevated at 2.22 µmol/L. After 7 months, his heart function had improved to within the normal range, with fractional shortening of 33% and an ejection fraction of 63%, with plasma C3DC level at 1.53 µmol/L. At 9‐months of treatment, urine malonate and methylmalonate excretion had decreased to 150.11 and 15.11 mmol/mol Cr, respectively. Currently, the patient is 5 years old and is continuing dietary intervention, L‐carnitine, and carvedilol. He has been growing normally (height in the 25–50th percentile; weight in the 50–75th percentile; head circumference in the 50–75th percentile) without any instances of hypoglycemia, seizure, or metabolic crisis. However, he has shown an overall developmental delay (developmental age of 1.3 years and social age of 2.7 years, as assessed by the Psychoeducational Profile Revised and the Social Maturity Scale, respectively), and requires speech and physical therapy, even though no abnormal focal lesions were found in his brain MRI. Last biochemical exam at the age of 4‐year 7‐months showed much improvement in the levels of plasma C3DC carnitine (0.80 µmol/L), urine malonate (47.9 mmol/mol Cr), and urine methylmalonate (11.3 mmol/mol Cr).

### 2.3 Results of molecular genetic analysis

**Figure 1:**
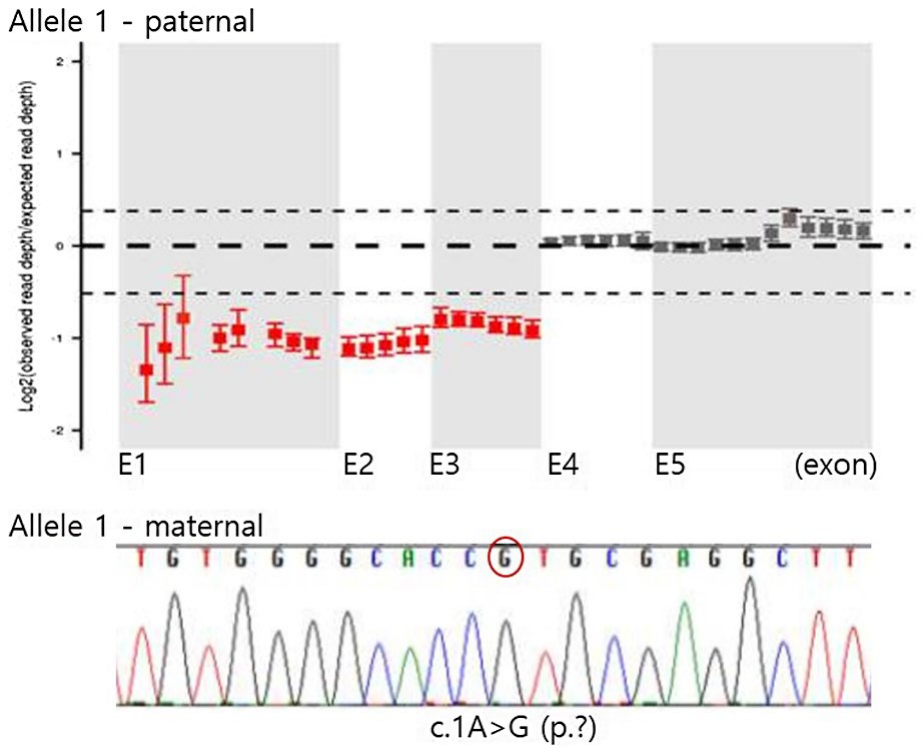
A partial sequence of cloning alleles of the *MLYCD* gene (NM_012213.3) showing a missense mutation at nucleotide 1 of the start codon (c.1A>G) in one allele, and deletion of exons 1–3 in the other allele, as identified using quantitative PCR

To understand the genetic basis of the clinical manifestations described above, we performed direct sequencing of the *MLYCD* gene (NM_012213.3) and quantitative PCR of each coding exon. Direct sequencing analysis identified a novel pathogenic mutation in our patient, c.1A>G. A partial deletion encompassing exon 1, 2, and 3 was also detected using quantitative PCR to screen copy number variation in *MLYCD*. Family screening revealed that his mother is a heterozygous carrier of the c.1A>G mutation and his father has a heterozygous exon 1–3 deletion. Therefore, the observed MCD deficiency was confirmed to be caused by compound heterozygous pathogenic mutations in *MLYCD,* the c.1A>G mutation, and an exon 1–3 deletion (Table 2; Figure 1).

**Table 2:** Results of the *MLYCD* gene analysis of the patient and his parents

	Allele 1	Allele 2
Patient	c.1A > G (p.?), hemizygote	exons 1–3 deletion, heterozygote
Father	−	+
Mother	+ (heterozygote)	−

Note

Reference Sequence: NM_012213.3.

## 3 DISCUSSION

Until the early 21st century, there were no adequate methods to screen MA in the preclinical stage (Pourfarzam, Zadhoush, & Sadeghi, 2016). However, since 2003, early cases of MA have been diagnosed in the neonatal period owing to the introduction of nationwide newborn screening programs in many countries (Santer et al., 2003).

MA is extremely rare with an estimated prevalence of less than 1 in 400,000 newborns occurring worldwide (Lee et al., 2017; Wang et al., 2019). In Korea, from 2006 to 2015, only six infants have been suspected of having MA as shown by the results of first tier newborn screening using TMS(Lee et al., 2017; Santer et al., 2003), resulting in a detection rate of 1/574,206. Interestingly, newborn screening using TMS performed during the neonatal period failed to detect biochemical abnormalities in our patient. When the test was repeated at 3 months of age, it showed an elevated C3DC level, leading to a clinical diagnosis of MA. Our case emphasizes that if inherited metabolic disease is clinically suspected, repeated TMS with corroborative biochemical studies, including urine organic acid analysis, may be beneficial for diagnosis.

Approximately 50 molecular variations related to the *MLYCD* gene have been classified as pathogenic or likely pathogenic, according to the ClinVar database (https://www.ncbi.nlm.nih.gov/clinvar). However, the correlation between these genetic variants and resulting phenotypes has not been elucidated. Our patient exhibited a novel pathogenic variant, c.A> G, in the start codon of *MLYCD*. This variant was detected using Sanger sequencing for all coding exons of the gene. Because the chromatogram showed a homozygous c.1A>G change, we also studied this variant in his parents to verify segregation. Interestingly, his mother harbored the c.1A>G variant heterozygously, whereas his father did not. Therefore, we continued our analysis using quantitative PCR of each coding exons and detected a previously reported heterozygous exon 1–3 deletion inherited from his father (Liu et al., 2016).The phenotypes exhibited by our patient were more severe than those previously reported for a pediatric patient in China who also had an exon 1–3 deletion. The Chinese patient, who mainly exhibited poor performance at school, without cardiomyopathy or metabolic acidosis, was not diagnosed until the age of 9. The C3DC level and urine malonate excretion at diagnosis were also much higher in our patient. On the other allele, the novel mutation found in our patient is located in the start codon (c.1A>G) and is classified as a null variant, while the mutation in the Chinese patient was a missense variant, c.911G>A (p.Gly304Glu) in exon 4. The nature of the mutation on the other allele may explain the differences observed in phenotypes and disease severity between the two patients with the exon 1–3 deletion.

Our patient initially presented with DCMP and a decreased ejection fraction of 20%, requiring digoxin therapy at 3 months of age. After 2 weeks of medical therapy, the ejection fraction did not improve significantly. Following biochemical diagnosis of MA, a low‐fat and high‐carbohydrate diet with MCT and L‐carnitine supplement was administered. This dramatically improved his cardiac function which reached ejection fractions of 43% and 63% at 3‐weeks and 7‐months of dietary intervention, respectively. Improvement of cardiac function following dietary intervention was consistent with previous MA cases exhibiting cardiomyopathy (Ficicioglu et al., 2005; Footitt et al., 2010).

Our patient is now 5 years old and his cardiac function remains stable; he is currently being treated with dietary intervention with L‐carnitine and beta‐blocker. He shows good clinical performance with normal growth profiles in parameters of height (25–50th percentile), weight (50–75th percentile), and head circumference (50–75th percentile). However, his developmental age was estimated at only 2.8 years old, when he was assessed using the Psychoeducational Profile Revised and Receptive and Expressive Vocabulary Test. Developmental delay in MA patients may be explained by the neurotoxic effects of accumulating malonyl‐CoA esters and neonatal hypoglycemic events, although the mechanism is not fully understood (Xue et al., 2012).

Early diagnosis of MA (3 months old) enabled timely dietary interventions and reversed the deterioration of cardiac function with improvement of biochemical abnormalities in our patient. MA can be detected by newborn screening before the onset of signs or symptoms; however, negative newborn screening results do not necessarily exclude the possibility of disease. As early diagnosis and treatment is able to prevent or minimize progressive and life‐threatening complications in MA and other metabolic myopathies, active metabolic screening to identify manageable causes is highly recommended, especially for infantile cardiomyopathies.

## CONFLICT OF INTEREST

The authors declare no conflict of interest.

## AUTHOR CONTRIBUTIONS

S.H.L., data assembly and manuscript preparation; M.K.S., clinical analysis about cardiomyopathy; J.S., biochemical analysis; K.S.P., molecular genetic analysis; J.M.K., design of this study.

## Data Availability

The data that support the findings of this study are available from the corresponding author upon reasonable request.
